# Effects of Essential Oil Derived from the Bitter Orange (*Citrus aurantium*) on Growth Performance, Histology and Gene Expression Levels in Common Carp Juveniles (*Cyprinus carpio*)

**DOI:** 10.3390/ani11051431

**Published:** 2021-05-17

**Authors:** Ümit Acar, Osman Sabri Kesbiç, Sevdan Yılmaz, Burak Evren İnanan, Fahriye Zemheri-Navruz, Funda Terzi, Francesco Fazio, Vincenzo Parrino

**Affiliations:** 1 Bayramiç Vocational School, Çanakkale Onsekiz Mart University, Çanakkale 17100, Turkey; 2 Department of Microbiology, Veterinary Faculty, Kastamonu University, Kastamonu 37200, Turkey; osmankesbic@yahoo.com; 3 Department of Aquaculture, Faculty of Marine Sciences and Technology, Çanakkale Onsekiz Mart University, Çanakkale 17100, Turkey; sevdanyilmaz@comu.edu.tr; 4 Department of Veterinary Science, Eskil Vocational School, Aksaray University, Aksaray 68000, Turkey; burak_inanan@hotmail.com; 5 Department of Molecular Biology and Genetics, Faculty of Science, Bartın University, Bartın 74100, Turkey; fahriyezmhr@hotmail.com; 6 Department of Pathology, Faculty of Veterinary Medicine, Kastamonu University, Kastamonu 37200, Turkey; fundaterzi@kastamonu.edu.tr; 7 Department of Veterinary Sciences, University of Messina, 98168 Messina, Italy; ffazio@unime.it; 8 Department of Chemical, Biological, Pharmaceutical, and Environmental Sciences, University of Messina, 98168 Messina, Italy; vparrino@unime.it

**Keywords:** gene regulation, growth performance, common carp, neroli oil, bitter orange

## Abstract

**Simple Summary:**

The aim of this analysis was to reveal the effects of *Citrus aurantium* essential oil on growth performance, histopathological effect on digestive system organs, expression levels of some growth-related genes in the muscle tissue and some immune-related genes in the head kidney of common carp *Cyprinus carpio*. To determine the effect of essential oil on carp, experimental diets containing different concentrations of essential oil were prepared, and the fish were fed these diets for 60 days. At the end of the feeding experiment, it was observed that low-dose essential oil supplementation increased immune related gene expression levels and growth-related gene expression levels in head kidney and muscle tissue, respectively. Pathological findings were observed in the liver and intestines of fish fed with high doses of essential oil supplemented diets.

**Abstract:**

The aim of this study was to detect effects of bitter orange (*Citrus aurantium*) essential oil, commonly called neroli oil (NO) (0, 0.25, 0.50, 1, and 1.5% referred to as NO_0_ NO_0.25_, NO_0. 05_, NO_1_ and NO_1.5_, respectively) on growth performance output and expression levels of some growth-related genes in the muscle tissue and some immune-related genes in the head kidney and pathological differences in digestive system organs of common carp *Cyprinus carpio*. The NO_0.25_ group had a large improvement in growth efficiency at the end of the 60-day feeding cycle. Real-time PCR (Bio RAD, USA) system was used to detect variations in gene expression levels. Furthermore, NO supplementation of up to 0.25% in muscle tissue controlled the release of growth hormone (GH) and insulin-like growth factor I (IGF-I). Furthermore, in the NO_0.25_ treatment category, immune response gene levels TNF-α, IL-8 and IL-1ß increased in head kidney tissue. In the histological examination of the liver and intestine, there were significant differences between fish fed with N_1_ and N_1.5_ diets. This study confirms that dietary supplementation of NO up to 0.25% can improve common carp growth efficiency and increase the expression of genes (GH and IGF-I) related to muscle growth, TNF-α, IL-8 and IL-1ß genes related to immune status, and liver and intestine histological status of common carp.

## 1. Introduction

Regarding the advancement of intensive aquaculture systems, one of the most critical issues of aquaculture has been to protect the fish from diseases while harvesting the maximum amount of product per farm unit. Researchers believe that improving fish feed quality could be the most efficient approach to this issue, and there has been research into high performance feeds for a long time. As a consequence, several synthetic feed additives, especially antibiotics, have been applied to fish feeds to protect the feed quality, and protect fish from potential diseases and environmental problems [1]. However, it has been identified that the long-term application of antibiotics and related synthetics leads to the development of antibiotic-resistant microorganisms [1]. Because of the implementation of good aquaculture practices, growing nutritional knowledge and industrial applications, study of feed supplements has centered on the use of plant extracts in aquafeeds over the past decades [2,3,4]. Extracts collected from various sections of different citrus species inhibit oxidative stress [5], increase development [6] and strengthen the immune system [7] in fish, according to studies performed with the use of plant extracts in fish feeding.

The basic oil derived from the bitter orange (*Citrus aurantium*) blossom is known as neroli oil (NO) [8]. The oil is used in traditional folk medicine throughout the world, especially for its sedative effect as a nontoxic plant-based extract [9]. The US Food and Drug Administration (FDA) finds essential NO to be generally recognized as safe (GRAS) for internal usage due to its nontoxic quality [10]. The volatile component profile of NO includes several molecules, especially limonene [11]. It has been determined that NO has several beneficial effects, especially in laboratory animals, due to the bioactive molecules it contains. NO was shown to have antianxiety effects in rats by regulating serotonin receptors, and an antidepressant effect in mice [12]. NO has also been shown to have sedative, antianxiety and antidepressant properties in mice. Although the metabolism of the sedative effect observed in mice has not yet been fully understood, it is thought that the NO effect on sedation is due to its major component linalool. [13]. The majority of previous experiments have reported beneficial effects like growth promotion, immune boosting and disease resistance of different fish species utilizing other citrus species products such as sweet orange peel [7], lemon peel [14] and bergamot oil [6].

In recent years, advanced molecular techniques have provided greater opportunities for a better understanding of the metabolism and function of nutrients. Due to the promising results found in laboratory animal studies, and the rich bioactive content of NO, this research aimed to investigate the effects of NO on common carp, one of the most commonly developed breeding organisms in the world.

## 2. Materials and Methods

### 2.1. Fish and Experimental Conditions

The experimental analysis used 450 common carp collected from the Mediterranean Fisheries Research, Production, and Training Institute in Antalya, Turkey. The experimental fish were acclimatized in a 1000 L tank for 14 days and fed with commercial carp feed during acclimatization period. During the acclimatization and experimentation periods, the fish were held at ambient temperatures of 25 ± 1 °C, dissolved oxygen of 6.02 ± 0.78 mg L^−1^ and pH of 7.35 ± 0.68. In this experiment, 450 fish (1.94 ± 0.05 g) were randomly assigned to one of five groups (15 aquaria) and assigned the codes NO_0_, NO_0.25_, NO_0.50_, NO_1_ and NO_1.5_. Each treatment was formed in triplicate. For 60 days, the fish were fed twice a day at 9:00 and 16:00

### 2.2. Experimental Diets

Experiment diets were generated by using 0.25%, 0.50%, 1% and 1.5% NO. A non-NO-supplemented control diet was prepared, and chemical composition of test diets analyzed using [15] guidelines (Table 1). The dry ingredients for the diet preparation weremixed in a laboratory food blender. To provide a suitable pulp, the mixtures were primed with tap water. The wet materials were used to make 1-mm pellets, which were then dried at 40 °C in a drying cabinet and kept at −20 °C before feeding.

### 2.3. Determination of Aromatic Components in Neroli Oil

The aromatic components of NO were measured using Chromatography-Mass Spectrometry (GC-MS) after it was obtained from a nearby medicinal herb shop and diluted with NO high-purity ethanol at a ratio of 1/20. (Shimadzu GCMS QP 2010 ULTRA). By comparing the Wiley W9N11 spectra libraries, ion chromatograms were established. Table 2 indicates the derived phytochemicals, their proportional percentages in the NO, and their retention periods. The major compounds in NO were identified using GC. Linalyl acetate (42.77%) was the most prevalent chemical constituent, followed by linalool (27.41%) and geranly acetate (10.21%); limonene dioxide (3.50%) was also found.

### 2.4. Calculation of the Growth Performance of Fish

The fish were individually weighed at the start and end of the experiment. Fish growth performance was estimated using following equations:

Specific growth rate (SGR, %/day) = 100 (ln final fish weight) − (ln initial fish weight)/experimental days(1)

Feed conversion ratio (FCR) = feed fed/weight gain(2)

### 2.5. Histopathological Examination

Systemic necropsy of carp fish was used to evaluate the liver and intestines in a 10% formaldehyde solution. After that, the tissues were cut and moved to cassettes. Routine pathology follow-up was done after the cassettes were cleaned under running water, and paraffin was blocked. Hematoxylin-eosin staining was conducted on 5 µ thick pieces of paraffin blocks cut in a rotary microtome (Thermo Scientific HM 340 E, Walldorf, Germany) and mounted on adhesive slides covered with a coverslip [16]. A light microscope was used to inspect and image the sections. Histopathological changes, degree and extent of change were assessed as - (0): none, + (1): mild occurrence, + + (2): moderate occurrence, + + + (3): severe occurrence, according to previously studies [17,18].

### 2.6. Total RNA Isolation and Quality Control

For the immune-related gene expression analysis, head kidney tissue, and for the growth-related gene expression analysis, muscle tissue, from five fish from each tank were dissected and deposited in RNAlater solution at −20 °C for complete RNA extraction. RNA was extracted from head kidney and muscle tissue samples using the GeneJet RNA purification kit (Thermo Science, Waltham, MA, USA). The samples were then placed in RNAlater solution (Thermo Scientific, USA). The quality of isolated RNA was assessed using a MultiskanTM FC Microplate Photometer (Thermo Scientific, USA).

### 2.7. Primer Design and cDNA Synthesis

DNase-I (Thermo Scientific, USA) was used to distinguish DNA from RNA, and the RevertAid H Minus Single Strand cDNA Synthesis Kit was used to build cDNA (Thermo Scientific, USA). We used the NCBI website, mRNA sequences of *β*-actin, *TNF*-α, *IL-1ß*, *IL-8*, *GH* and *IGF-1* genes that are specific to *Cyprinus carpio*, and a computer package software called FastPCR 6.0 to develop primers [19]. Table 3 shows the primer sequences, complete base volume and gene bank amounts.

### 2.8. Real-Time PCR Analysis

A real-time PCR (Bio RAD, Hercules, CA, USA) system was used to detect variations in gene expression levels across study classes. The PCR study was carried out using the PCR blend, Maxima SYBR Green qPCR Master Mix and ROX Solution (Thermo Scientific, Waltham, MA, USA) [20].

### 2.9. Identification of Gene Expression Levels

The CFX Manager 3.1 program was used to analyze the real-time PCR performance. Proportional shifts in mRNA expression levels of target genes were determined using the 2- Ct procedure, which is based on cycle thresholds (Ct) of amplification curves obtained during the amplification phase, which included denaturation, primer annealing and chain extension stages [20].

### 2.10. Statistical Analysis

In this study, one-way analysis of variance (One-Way ANOVA) and the Tukey test were conducted to find differences and averages among experimental groups in terms of each gene according to different gender groups. SPSS 15.0 for the Windows package program was used in analyzing data.

## 3. Results

### 3.1. Growth Performance

Table 4 shows the effects of NO on the growth performance of common carp (*C. carpio*). In terms of growth performance, there was a large gap between the study groups at the end of the experiment. It was discovered that an improvement in NO in the ration diminished fish growth efficiency. The fish fed NO_0.25_ had the highest growth efficiency, which was found to be statistically distinct from the control group.

### 3.2. Histopathological Results

Histopathological semiquantitative scoring results of liver and intestinal tissue are summarized in Table 5.

#### 3.2.1. Liver

**Figure 1:**
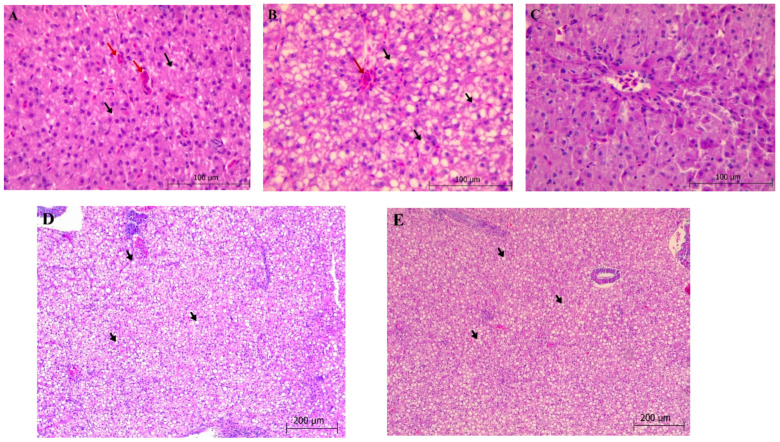
Histological preparations of livers (ematoxylin-eosin staining) of fingerling common carp after feeding experimental diets with graded levels of neroli oil (0–1.5%) for 60 days. (**A**) Degeneration of hepatocytes (black arrows) and congestion of sinusoids (red arrows) (N_0_). (**B**) Hydropic degeneration (black arrows) and fat vacuoles in hepatocytes and congestion (red arrow) (NO_0.25_). (**C**) Degeneration, fat vacuoles in hepatocytes and congestion (NO_0.50_). (**D**) Hydropic degeneration and steatosis/lipid deposit in hepatocytes (black arrows) (NO_1_). (**E**) Hepatocytes are hydropic and bubble-like degeneration, steatosis (black arrows) (NO_1.5_).

Balloon-like and hydropic degeneration in hepatocytes, nuclear pycnosis, vacuolar degeneration, congestion in sinusoids and accumulation of fatty vocals and steatosis were evaluated histopathologically and scored semiquantitatively. Diffuse balloon-like and hydropic degeneration in hepatocytes was found to be more severe (*p* > 0.05) in the groups where 0.25% and 1.5% NO was used (Figure 1B–E). Accumulation of diffuse fat vacuoles and severe fat in the liver were determined in the groups where the highest rate of NO was used (Figure 1D,E), while it was found to be similar in the control group and the group in which low amounts of NO were used (Figure 1A–C). Pycnosis in hepatocytes, and congestion in sinusoids and Kupffer cells were found in all experimental groups. In addition, no hyperplasia was found in the bile ducts.

#### 3.2.2. Intestine

**Figure 2:**
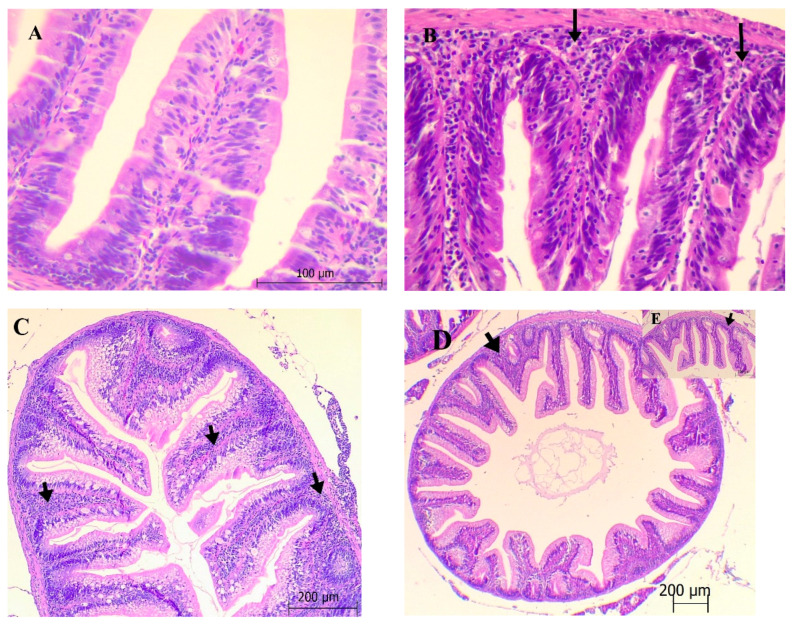
Histological preparations of intestine (ematoxylin-eosin staining) of fingerling common carp after feeding experimental diets with graded levels of neroli oil (0–1.5%) for 60 days. (**A**) Lamina propria cell infiltration and hyperemia in the submucosa (NO_0.25_). (**B**) Cell infiltration (black arrows) in the submucosa (NO_0.50_). (**C**) Cell infiltration in the lamina propria and submucosa (black arrows) and hyperemia in the submucosa (NO_1_). (**D**,**E**) Lamina propria, severe cell infiltration in the submucosa (black arrows) and hyperemia (NO_1.5_).

Cell infiltration and hyperemia were the most common changes in the lamina propria and submucosa. Cell infiltration in the lamina propria and submucosa was found to be more severe (+3) in the groups (*p* < 0.05) in which NO was used in higher doses (Figure 2C–E) compared to low doses of NO (Figure 2A,B). Congestion was determined in all groups. In addition, it was determined that there was no degeneration and necrosis in the lamina epithelialis.

### 3.3. Expression of Growth and Immune Related Genes

**Figure 3:**
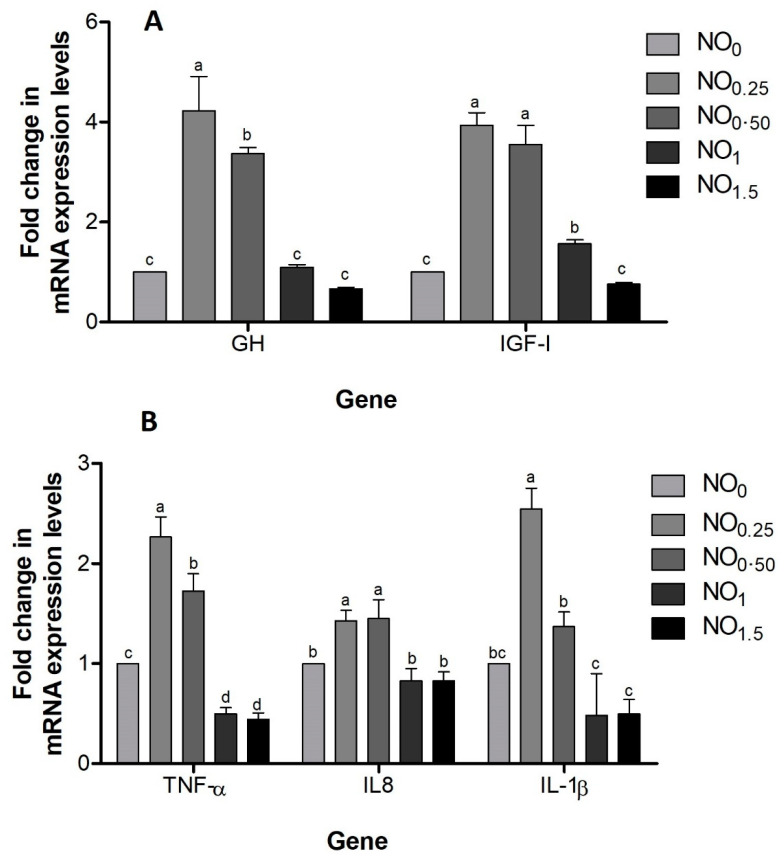
Expression of growth hormone (GH) and insulin growth factor I (IGF-I) in the muscle (**A**) and TNF-α, IL1-β and IL-8 in the head kidney (**B**) of common carp fed on diets with 0 (control), 0.25%, 0.50%, 1.0% and 1.5% neroli oil (NO). *n* = 6 X ± SD. a,b,c Within a row mean values with different letters are significantly different from the other groups (*p* < 0.05).

Expression profiles of immune related genes (*TNF-α*, *IL-1β* and *IL-8*) and growth related genes (*GH* and *IGF-1*) were examined in head kidney and muscle tissue (Figure 3) at the end of the experiment. Transcription levels of *TNF-α*, *IL-1β*, *IL-8*, *GH* and *IGF-1* were slightly upregulated in NO_0.25_ and NO_0.50_-supplemented diets (Figure 3). However, significant differences were observed in the expression of all immune and growth-related genes in fish head kidney and muscle tissue fish fed with NO_1_ and NO_1.5_ diets.

## 4. Discussion

The use of chemicals in diets as immunostimulants or growth promotors can be harmful to animals, consumers and the environment [21]. That is why research is focused on natural products to replace chemicals in fish feeds. For this purpose, this study aimed to determine the possibilities of using NO obtained from bitter orange (*Citrus aurantium*) in carp (*Cyprinus carpio*) diets and to determine its effects on growth performance, gene expression and liver and intestinal histology. The results indicate that dietary NO levels greater than 1% have a negative impact on carp development. Similarly, dose dependent effects of pomegranate seed (*Punica granatum)* EOs in rainbow trout diets were recorded, where 5% and 10% of pomegranate seed oils (*P. granatum*) in diets stimulated growth production while 20% significantly suppressed growth [22]. Mehrabi [23] discovered that symbiotic bacteria in the intestine play an important role in digestion, and that an increase in the population of commensal bacteria can affect growth efficiency. It should be expected that utilizing NO up to 1% in fish diets would affect the microbial equilibrium in the digestive system and would have a negative effect on fish growth efficiency. These findings further highlight the significance of appropriate dosing in achieving the desired outcomes [24]. There are many advantages to including medicinal plants in fish, but there are often dangers from active additives and overdosing. However, at the required dose, they should not trigger any complications [25].

Histopathological experiments play an important role in exposing improvements in fish tissues and cells during adverse circumstances [26,27]. Antioxidant, anti-inflammatory, and essential oils derived from plants are used as feed additives because of the characteristics in the fish diets [28,29,30]. The liver is largely responsible for lipid metabolism in fish, which involves both fatty acid production and oxidation. Yilmaz [31] discovered that high-fat diets trigger liver lipoid degeneration (steatosis) in carp (*Cyprinus carpio*). In addition, hepatocyte cytoplasmic vacuolation has been observed in the livers of rainbow trout fed with *Origanum onites* essential oil-supplemented (2.5 mL kg^−1^) diets for 90 days [28]. The liver histopathological examination in the current study did not reveal any significantly differences in lesions other than lipid vacuole accumulation between the experimental groups (*p* > 0.05). On the other hand, significantly higher lipid vacuole accumulation was observed in the livers of trout fed with NO_1_ group diets than fed with control and NO_0.25_ (*p* < 0.05).

Intestinal anatomy represents fish wellbeing in terms of nutrient processing ability and immunological functions [32]. Brum [33] discovered that clove (*S. aromaticum*), basil (*O. gratissimum* L.), and ginger essential oil (*Zingiber officinale*) had greater lymphocytic penetration in the intestine of fish fed 1.5% ginger essential oil, and that essential oils had harmful consequences at higher doses. The oils studied, on the other hand, have been shown to boost the immune system and reduce tissue harm induced by *Streptococcus agalactiae* infection. In the groups that used NO in the study’s histopathological analysis of the intestines, cell invasion and hyperemia were present in the lamina propria and submucosa. Cell infiltration in the lamina propria and submucosa was observed to be more serious in the groups that used higher doses of NO (*p* < 0.05) relative to the groups that used low doses of NO. These histopathological changes in the intestines were thought to cause inflammation when large doses of NO were used, and they could be due to the toxicity or allergic disorder of such oil components.

Changes in muscle development in fish are a biological reality affected by both external and endogenous hormonal influences, and fish weights correlate with high mRNA expression of *IGF-1* and *GH* in fish under the same conditions [34,35]. Muscle development is inversely proportional to fish growth. In this study, the gene expression showed upregulated *GH* and *IGF-1* results in carp muscle tissue. A previous study conducted by [36] reported high levels of *IGF-1* in liver tissue fish fed with 400 and 600 ppm limonene-supplemented diets. As a different finding, our results showed decrease in *GH* and *IGF-1* expression in muscle tissue due to increasing NO levels in diets. This suggests that using 0.25 percent NO in diets enhances the potency of NO and improves the common carp’s growth efficiency in a dose-dependent manner. Similarly, changes in gene expression levels depending on the dose were reported by [37] when common carp were fed with olive leaf extract. In animals, cytokines are markers of inflammation, and stress causes inflammation in fish [38]. The present results clearly showed that dietary NO had anti-inflammatory effects on common carp fed with 0.25% supplemented diets as an indicator of boosted health. In general, the incorporation of immunostimulants such as essential oil or extract in diets of different fish species can beneficially improve the expression of *IL-8 IL-1β* and *TNF-α* [39,40,41]. These findings are not surprising, since antioxidant compounds in herbal products have been shown to shield cells from the adverse effects of reactive oxygen species such as superoxide, peroxyl radicals, hydroxyl radicals and peroxynitrite [42].

## 5. Conclusions

In conclusion, the current findings show that including NO in diets at a level of 0.25% for common carp improves growth performance, growth related gene expression levels in muscle, and immune related gene expression levels in spleen tissue without affecting liver or intestine histomorphology. Therefore, more studies are required to understand the effects of NO on digestive enzymes activity or molecular mechanisms of fish.

## Figures and Tables

**Table 1:** Percentage and proximate composition of the experimental diets supplemented with different percentages of neroli oli (NO).

Ingredients (%)	NO_0_	NO_0.25_	NO_0.50_	NO_1_	NO_1.5_
Fish meal	23.00	23.00	23.00	23.00	23.00
Soybean meal	37.00	37.00	37.00	37.00	37.00
Wheat meal	12.00	12.00	12.00	12.00	12.00
Fish oil	5.00	5.00	5.00	5.00	5.00
Vitamin-mineral mix	4.00	4.00	4.00	4.00	4.00
Corn starch	19.00	18.75	18.50	18.00	17.50
Neroli oil	0.00	0.25	0.50	1.00	1.50
Proximate composition (DM%)					
Crude protein	34.41	34.33	34.58	34.49	34.43
Crude lipid	7.80	7.71	7.88	7.91	7.95
Crude ash	5.65	5.49	5.80	5.86	5.79

**Table 2:** Volatile compounds of neroli oil.

	Compounds	Retention Time	Concentration %
1	Linalool	15.883	27.41
2	α- Terpineol	19.248	3.26
3	Linalyl acetate	21.725	42.77
4	Geranial	22.137	0.64
5	Geranyl acetate	25.369	10.21
6	Linalool 8-monooxygenase	27.032	0.80
7	Limonene dioxide	29.949	3.50
8	Indanedione	30.450	1.25
Total identified volatile content value			89.84

**Table 3:** Primer sequences used in this study.

Gene	Oligonucleotide Sequence		Product Size (bp)	Gene Bank No.
*β-Actin*	F	CTGGTATCGTGATGGACTCT	204	M24113
	R	CAGAGCTTCTCCTTGATGTC		
*TNF-α*	F	GTGTCTACAGAAACCCTGGA	109	AJ311800
	R	AGTAAATGCCGTCAGTAGGA		
*IL-1ß*	F	TTACAGTAAGACCAGCCTGA	89	AJ245635
	R	AGGCTCGTCACTTAGTTTGT		
*IL-8*	F	GTCTTAGAGGACTGGGTGTA	120	AB470924.1
	R	ACAGTGTGAGCTTGGAGGGA		
*GH*	F	TCTTCGCATCTCTTTTCACC	210	M27000.1
	R	AGTCGGCCAGCTTCTCA		
*IGF-1*	F	GGCATTGGTGTGATGTCTTT	96	KP661168.1
	R	CATATCCTGTCGGTTTGCTG		

**Table 4:** Growth performance of common carp fed for 60 days with experimental diets supplemented with different percentages of neroli oli (NO).

	NO_0_	NO_0.25_	NO_0.50_	NO_1_	NO_1.5_
Initial weight (g)	1.88 ± 0.05	1.96 ± 0.03	1.91 ± 0.05	1.97 ± 0.03	1.95 ± 0.02
Final weight (g)	5.76 ± 0.10 ^c^	6.66 ± 0.20 ^a^	6.18 ± 0.15 ^b^	5.81 ± 0.13 ^bc^	5.33 ± 0.06 ^d^
Relative growth rate (%)	206.5 ± 2.87 ^bc^	235.4 ± 10.11 ^a^	223.2 ± 1.26 ^ab^	193.7 ± 8.00 ^c^	173.8 ± 5.26 ^d^
Specific growth rate (% day^−1^)	2.49 ± 0.02 ^bc^	2.71 ± 0.07 ^a^	2.60 ± 0.01 ^ab^	2.39 ± 0.06 ^c^	2.23 ± 0.04 ^d^
Feed conversion ratio	0.86 ± 0.01 ^b^	0.71 ± 0.03 ^d^	0.78 ± 0.02 ^c^	0.87 ± 0.03 ^b^	0.98 ± 0.02 ^a^

*n* = 3 X ± SD. ^a–d^ Within a row mean values with different letters are significantly different from the other groups (*p* < 0.05).

**Table 5:** Histopathological observation on liver and intestine of common carp fed for 60 days with experimental diets supplemented with different percentages of neroli oil (NO).

Organs	Lesion	NO_0_	NO_0.25_	NO_0.50_	NO_1_	NO_1.5_
LIVER	Balloon-like and hydropic degeneration of hepatocytes	1.50 ± 0.54	2.16 ± 0.98	1.00 ± 0.00	1.66 ± 0.81	2.66 ± 1.03
	Lipid vacuole accumulation	0.66 ± 0.81 ^b^	0.66 ± 0.51 ^b^	0.75 ± 0.50 ^ab^	2.00 ± 1.09 ^a^	1.66 ± 0.51 ^ab^
	Pyknotic hepatocytes	0.16 ± 0.40	0.83 ± 0.75	0.25 ± 0.50	0.66 ± 0.81	0.83 ± 0.40
	Congestion/dilated sinusoids	0.83 ± 0.40	0.83 ± 0.75	0.75 ± 0.50	1.33 ± 0.51	1.16 ± 0.40
INTESTINE	Cell infiltration in lamina propria	0.50 ± 0.54 ^c^	1.00 ± 0.00 ^bc^	1.25 ± 0.50 ^bc^	2.20 ± 0.44 ^a^	1.50 ± 0.54 ^ab^
	Cell infiltration in submucosa	0.50 ± 0.54 ^c^	0.80 ± 0.44 ^bc^	1.75 ± 0.50 ^a^	1.80 ± 0.44 ^a^	1.66 ± 0.51 ^ab^
	Congestion	0.66 ± 0.51	0.40 ± 0.89	0.25 ± 0.50	0.80 ± 0.44	1.00 ± 0.63

*n* = 6 X ± SD. ^a–c^ Within a row mean values with different letters are significantly different from the other groups (*p* < 0.05).

## Data Availability

Not applicable.
